# Involvement of the Wnt/β-Catenin Signaling Pathway in the Cellular and Molecular Mechanisms of Fibrosis in Endometriosis

**DOI:** 10.1371/journal.pone.0076808

**Published:** 2013-10-04

**Authors:** Sachiko Matsuzaki, Claude Darcha

**Affiliations:** 1 CHU Clermont-Ferrand, CHU Estaing, Chirurgie Gynécologique, Clermont-Ferrand, France; 2 Clermont Université, Université d'Auvergne, ISIT UMR6284, Clermont-Ferrand, France; 3 CNRS, ISIT UMR6284, Clermont-Ferrand, France; 4 CHU Clermont-Ferrand, Service d’Anatomie et Cytologie Pathologiques, Clermont-Ferrand, France; Boston University Goldman School of Dental Medicine, United States of America

## Abstract

**Background:**

During the development and progression of endometriotic lesions, excess fibrosis may lead to scarring, chronic pain, and altered tissue function. However, the cellular and molecular mechanisms of fibrosis in endometriosis remain to be clarified.

**Objectives:**

The objective of the present study was to investigate whether the Wnt/β-catenin signaling pathway was involved in regulating the cellular and molecular mechanisms of fibrosis in endometriosis in vitro and to evaluate whether fibrosis could be prevented by targeting the Wnt/β-catenin pathway in a xenograft model of endometriosis in immunodeficient nude mice.

**Methods:**

Seventy patients (40 with and 30 without endometriosis) with normal menstrual cycles were recruited. In vitro effects of small-molecule antagonists of the Tcf/β-catenin complex (PKF 115-584 and CGP049090) on fibrotic markers (alpha smooth muscle actin, type I collagen, connective tissue growth factor, fibronectin) and collagen gel contraction were evaluated in endometrial and endometriotic stromal cells from patients with endometriosis. In vitro effects of activation of the Wnt/β-catenin signaling pathway by treatment with recombinant Wnt3a on profibrotic responses were evaluated in endometrial stromal cells of patients without endometriosis. The effects of CGP049090 treatment on the fibrosis of endometriotic implants were evaluated in a xenograft model of endometriosis in immunodeficient nude mice.

**Results:**

Treatment with PKF 115-584 and CGP049090 significantly decreased the expression of alpha smooth muscle actin, type I collagen, connective tissue growth factor and fibronectin mRNAs in both endometriotic and endometrial stromal cells with or without transforming growth factor-β1 stimulation. Both endometriotic and endometrial stromal cell-mediated contraction of collagen gels was significantly decreased by treatment with PKF 115-584 and CGP049090 as compared to that of untreated cells. The animal experiments showed that CGP049090 prevented the progression of fibrosis and reversed established fibrosis in endometriosis.

**Conclusion:**

Aberrant activation of the Wnt/β-catenin pathway may be involved in mediating fibrogenesis in endometriosis.

## Introduction

Endometriosis, a common cause of infertility and pelvic pain, is defined as the presence of endometrial glands and stroma in extra-uterine sites [1]. The prevalence of pelvic endometriosis approaches 6%–10% in the general female population; in women with pain, infertility, or both, the frequency is 35%–50% [1]. Histologically, endometriosis is characterized by dense fibrous tissue surrounding the endometrial glands and stroma [1]. During the development and progression of endometriotic lesions, excess fibrosis may lead to scarring, chronic pain, and altered tissue function, all of which are characteristics of this disease [2,3]. In particular, about 10%–15% of endometriosis cases are found to be more aggressive and tend to invade deep into the affected tissues and organs, forming dense scarring and producing more severe clinical symptoms, such as pelvic pain, dysmenorrhea, and dyspareunia [4]. Deep infiltrating endometriosis usually does not respond well to hormonal suppressive therapy, although endometriosis is an estrogen-dependent disease [4]. Adequate surgical excision of the lesions provides the best long-term results and symptomatic relief [4]. However, because of the deep invasive nature of the disease and the frequency of vital pelvic organ involvement, the gynecologist must be experienced and competent in performing bowel, bladder, and ureteral surgery [4].

Despite this knowledge, the cellular and molecular mechanisms of fibrosis in endometriosis remain to be clarified. Knowledge of these mechanisms is indispensable for the development of strategies to prevent and treat endometriosis.

Our previous study suggested that the Wnt/β-catenin signaling pathway may be aberrantly activated in endometriotic tissues and in the endometrium of patients with endometriosis during the mid-secretory phase [5,6]. We have recently demonstrated that cellular mechanisms known to be involved in endometriotic lesion development, cell proliferation, migration, and invasion of endometrial and endometriotic epithelial and stromal cells are inhibited by targeting the Wnt/β-catenin pathway in vitro [7]. Moreover, the Wnt/β-catenin pathway is involved in development, tissue self-renewal, and various diseases [8-11]. In addition, recent studies have demonstrated that activated Wnt/β-catenin signaling is involved in fibrosis in a number of organs [12-16]. Thus, we hypothesized that aberrant activation of the Wnt/β-catenin pathway may mediate the mechanisms of fibrogenesis in endometriosis. Further preclinical research is required to investigate whether inhibition of the Wnt/β-catenin signaling pathway may be effective in the prevention and treatment of endometriosis.

The objective of the present study was to investigate whether the Wnt/β-catenin signaling pathway was involved in regulating the cellular and molecular mechanisms of fibrosis in endometriosis in vitro and to evaluate whether fibrosis could be prevented by targeting the Wnt/β-catenin pathway in a xenograft model of endometriosis in immunodeficient nude mice.

## Materials and Methods

### Ethics statement

The research protocol was approved by the Consultative Committee for Protection of Persons in Biomedical Research (CCPPRB) of the Auvergne (France) region. Informed written consent was obtained from each patient prior to tissue collection. All animal care procedures followed were in accordance with the guidelines set by the European Communities Council Directive (86/609/EEC) and with French legislation on animal research. Institutional review board approval at the University of Auvergne was also obtained for the current animal study. The experiment was conducted under a license from the French Ministry of Agriculture.

### Patients

Patients aged 20–37 years undergoing laparoscopy for endometriosis were recruited at CHU Clermont-Ferrand for the present study. As controls samples, endometrial tissues were obtained from patients with uterine myomas who underwent laparoscopic myomectomy or patients who underwent laparoscopic surgery for tubal infertility. None of the women had received hormonal treatments, such as gonadotropin-releasing hormone agonists (GnRHa) or sex steroids, and none used intrauterine contraception for at least 6 months prior to surgery. Recruited patients had regular menstrual cycles (26–32 days) with confirmation of their menstrual history.

Samples from 40 patients who had histological evidence of pelvic endometriosis and samples from 14 patients with uterine myomas or samples from 16 patients with tubal infertility were used for the present analysis. Samples of tissue representing deep endometriotic lesions or ovarian endometriosis (ectopic endometrium) were paired with eutopic endometrial samples of the same patient for analyses. Deep infiltrating endometriosis was defined as endometriosis located 5 mm under the peritoneal surface. Patients with endometriotic ovarian cysts >3 cm in diameter were also included. Patients in which myomas had distorted the endometrial cavity were excluded. All of the patients with myomas in the present study had intramural and/or subserosal myomas. All of the patients with uterine myomas or tubal infertility had no endometriosis. The clinical characteristics of patients are shown in Table S1.

Endometrial tissue biopsies were performed just prior to surgery using an endometrial suction catheter (Pipelle, Laboratoire CCD, Paris, France). Samples of endometrial and endometriotic tissue were divided into two portions. The first tissue portion was fixed in 10% formalin-acetic acid and embedded in paraffin. The second portion was immediately collected in Hanks’ balanced salt solution (Life Technologies, Cergy Pontoise, France).

### Study design

The present study first investigated whether small interfering RNA (siRNA)-mediated knockdown of β-catenin, a key component of the Wnt signaling pathway, or small-molecule antagonists of the Tcf/β-catenin complex (PKF 115-584 and CGP049090), which disrupt the critical protein-protein interaction between β-catenin and Tcf, could decrease basal or transforming growth factor (TGF)-β1 stimulated expression of fibrotic markers in endometrial and endometriotic stromal cells. Next, small-molecule antagonists of the Tcf/β-catenin complex were evaluated for their ability to inhibit endometrial and endometriotic stromal cell-mediated collagen gel contraction. Then, we attempted to assess the effects of activation of the Wnt/β-catenin signaling pathway by treatment with recombinant Wnt3a on profibrotic responses in endometrial stromal cells of patients without endometriosis. Finally, we investigated whether fibrosis could be prevented by treatment with CGP049090 in a xenograft model of endometriosis in immunodeficient nude mice.

### Cell culture

Endometrial and endometriotic stromal cells were isolated as previously described [7]. Isolated cells were plated onto Primaria flasks (BD) in phenol red-free Dulbecco’s modified Eagle medium (DMEM)/F-12 containing 10% charcoal-stripped fetal bovine serum (FBS), 100 U/mL penicillin, 0.1 mg/mL streptomycin, and 0.25 µg/mL amphotericin B (Life Technologies, Cergy Pontoise, France) and incubated at 37 °C in 95% air/5% CO_2_. When the cells reached confluence, the first passages were used for experiments. Immunofluorescent staining was performed to determine the purity of the isolated endometrial and endometriotic stromal cells using monoclonal antibodies for human cytokeratin (MNF116, 1:100, DAKO, Glostrup, Denmark), vimentin (V9, 1:100, DAKO), factor VIII (1:100, DAKO), and CD45 (1:100, DAKO), as previously described [7]. The purity of the stromal cells was more than 98%, as judged by positive cellular staining for vimentin and negative cellular staining for cytokeratin, factor VIII, and CD45 [7].

### Treatment of the cells

Cells were seeded into 96-well plates (1 × 10^4^ cells per well) for cell proliferation analysis, 24-well plates (5 × 10^4^ cells per well) for quantitative real-time RT-PCR and immunocytochemistry, or 60-mm dishes (2 × 10^5^ cells per dish) for Wnt3a treatment in culture media. Cells were cultured at 37°C for 2 days and were then incubated for 24 h in serum-free culture media. Subsequently, the media were refreshed, and cells were treated with PKF 115-584 (6.25 µM) (Sigma-Aldrich, Lyon, France) or CGP049090 (6.25 µM) (Sigma-Aldrich), with or without TGF-β1 (5 ng/mL) (R&D Systems, Lille, France) or with Wnt3a (150 ng/mL) (R&D System) or TGF-β1 (5 ng/mL) (R&D Systems) in serum-free culture media.

### β-Catenin siRNA transfection

siRNA transfections were performed as previously reported in serum-free OPTI-MEM using 20 nM siRNAs and Lipofectamine [7]. Control siRNA (AM4611, Life Technologies) or validated human β-catenin siRNA (siRNA ID: s437; Life Technologies) were added to cells and incubated for 48 h. Subsequently, the media were refreshed with serum-free culture media, and cells were stimulated with TGF-β1 (5 ng/mL) (R&D System) or Wnt3a (150 ng/mL) (R&D System) for 24 h.

### Collagen gel contraction assay

Twenty-four-well culture plates were coated with 1% bovine serum albumin (BSA) and incubated for 1 h at 37°C to create a nonstick surface that prevented gels from attaching to the dishes. To investigate the effects of Wnt3a treatment on endometrial stromal cells, cells were treated for 72 h with Wnt3a (150 ng/mL) (R&D System) or vehicle. Endometriotic and endometrial stromal cells were trypsinized, counted, and seeded at a concentration of 2.5 × 10^5^ cells/mL into a 2.0-mg/mL Type I collagen solution (BD, Le Pont de Claix, France) in PBS containing 0.023 N NaOH. The collagen/cell suspension was vortexed, and 500 µL per well was added to the BSA-coated plates. The solution was allowed to polymerize for 60 min at 37°C. Five hundred microliters of culture media (2% charcoal-stripped FBS) containing PKF 115-584 (6.25 µM; Sigma-Aldrich), CGP049090 (6.25 µM; Sigma-Aldrich), or vehicle only was added to the 3-dimensional solidified collagen gels, and plates were returned to the incubator. For Wnt3a-treated endometrial stromal cells, 500 µL of culture media (2% charcoal-stripped FBS) was added. Collagen gel contraction was monitored over a period of 24 h, and the surface area of the contracted gels was measured at 0, 4, 6, 12, and 24 h using ImageJ software developed at the National Institute of Health. The percentage of FBS and the duration of the experiments were determined during preliminary experiments (Text S1). All experiments were performed in triplicate.

### RNA extraction

Total RNA was extracted using the Qiagen RNeasy Mini Kit according to the manufacturer’s instructions (Qiagen, Courtaboef, France). Briefly, after aspirating culture media completely, cells were lysed directly in the cell-culture plates. Then, lysates were mixed with an equal volume of 70% ethanol, and total RNA was purified using RNeasy mini spin columns. The eluted total RNA was stored at -80 °C until use. To eliminate potential genomic DNA contamination, RNA samples were treated with DNaseI (15 U; DNaseI, Qiagen) at room temperature for 15 min.

### Examination of RNA yield and integrity

RNA yield and integrity were analyzed using the RNA 6000 Pico kit and the Agilent Bioanalyzer 2100 (Agilent Technologies, Santa Clara, CA, USA). The RNA 6000 Pico kit allows determination of the integrity of very small amounts of RNA as well as estimation of the quantity of the isolated RNA, which has a linear range of 200–5,000 pg/µL. The RNA integrity number (RIN) value was >8.0 in all of the samples included in the present analysis using real-time RT-PCR [7,17,18].

### Quantitative real-time RT-PCR

mRNA expression of alpha smooth muscle actin (αSMA), type I collagen (Col-I), connective tissue growth factor (CTGF), fibronectin (FN) and Axin-2 as well as a non-Tcf/β-catenin or TGF-β1 target gene, hyaluronidase-2 (negative control), was measured by quantitative real-time RT-PCR with a Light Cycler as previously described [5,7]. PCR amplification was performed using the FastStart DNA Master SYBR Green I kit (Roche, Mannheim, Germany). Primer sets are shown in Table S2. Quantification of the targets in the unknown samples was performed using a relative quantification method with external standards. The target concentration was expressed relative to the concentration of a reference housekeeping gene, glyceraldehyde 3-phosphate dehydrogenase (GAPDH). After each run, a melting curve analysis was performed to verify the specificity of the PCR reaction. The procedure was repeated independently three times to ensure the reproducibility of the results. All of the samples with a cycle threshold (Ct) coefficient of variation value >5% were retested.

### Cell proliferation assay

Cell proliferation assays were performed 72 h after Wnt3a or vehicle treatment, using the CellTiter 96® AQueous One Solution Cell Proliferation Assay (MTS) (Promega, Charbonnières-les-Bains, France), as previously described [7]. All experiments were performed in triplicate.

### In vitro migration assay

In vitro migration assays were performed 72 h after Wnt3a or vehicle treatment using uncoated 24-well chambers/microfilters (BD), as previously described (7). Briefly, cells (5 × 10^4^ cells per chamber) in 500 µL phenol red-free DMEM/F-12 without FBS (Life Technologies) were seeded onto the upper chamber. In the lower chamber, 750 µL phenol red-free DMEM/F12 plus 10% charcoal-stripped FBS (Life Technologies) were added. Cell motility/migration was measured as the number of cells that migrated from a defined area of the uncoated microfilter through micropores in 24 h. All experiments were performed in triplicate. The micropore filters were stained with toluidine blue, and the number of cells that migrated through filters was counted in the entire area of each filter. To count cell numbers objectively, a computerized image analysis system consisting of a light microscope (Leica, Lyon, France; 20× objective, 10× ocular) and a color charge-coupling device camera (Sony, Paris, France) were utilized.

### Immunocytochemistry

Immunocytochemistry was performed 24 h after Wnt3a (150 ng/mL) (R&D System), TGF-β1 (5 ng/mL) (R&D System), PKF 115-584 (6.25 µM) (Sigma-Aldrich), CGP049090 (6.25 µM) (Sigma-Aldrich), or vehicle treatment. All experiments were performed in duplicate. Cells were then fixed for 10 min in 3.7% formaldehyde and permeabilized with 0.1% Triton X-100 for 5 min. Cells were then incubated with phalloidin conjugated to Alexa-fluor594 (Life Technologies) for 20 min for staining of f-actin or primary antibody (anti-αSMA, 1:200, Merck Millipore, Molsheim, France) for 1 h at room temperature. Following primary antibody incubation, cells were washed with PBS and incubated for 1 h in a 1:20 dilution of fluorescein-labeled anti-mouse antibodies (DAKO) in the dark. Cell nuclei were stained with 4′,6-diamidino-2-phenylindole (DAPI). Images were obtained using a FLoid Cell Imaging Station (Life Technologies). For quantification of αSMA-positive stromal cells, labeled cells were counted and reported as a percentage of the total number of DAPI-stained nuclei. To avoid a selection bias, all of the cells were evaluated, and the mean percentage of labeled cells was calculated for each specimen.

### Mouse model for endometriosis

#### a) Animals

Studies were conducted in adult (7–8 weeks old, 23–25 g) female Swiss nude mice (Iffa-Credo, Lyon, France). Mice were maintained in a barrier unit in a well-controlled, pathogen-free environment with regulated cycles of light/dark (12 h/12 h, 23–25°C) and allowed a 2-week period of acclimation to the vivarium before any procedures were performed. Mice had free access to food and water. After completion of the experiment, all mice were euthanized with an anesthetic overdose.

#### b) Development of mouse model for endometriosis

Nude mice were implanted subcutaneously (s.c.) in the neck region with a pellet of 17β-estradiol (0.72 mg/60-day release; Innovative Research of America, Sarasota, FL). Proliferative-phase human endometrial tissues were obtained from a total of 20 patients without endometriosis. Human endometrial tissue was washed with sterile serum-free DMEM/F-12. A total of 80 nude mice received a single injection of 10 proliferative endometrial fragments (1–2 mm^3^) in 200 μL of serum-free DMEM/F-12. S.c. injections were performed at a ventral midline site using 1-mL syringes and 18-gauge needles. All procedures were performed under isoflurane anesthesia.

#### c) Treatment with a small-molecule antagonist of the Tcf/β-catenin complex (CGP049090)

To investigate the time course of fibrosis development in the present animal model, endometrial tissues from a total of 10 patients without endometriosis were implanted into 4 mice simultaneously at day 0, and mice were then sacrificed on days 7, 14, 21, and 28 (Figure S1A). To investigate the effects of CGP049090 (Sigma-Aldrich) treatment on fibrosis, endometrial tissues from a total of 10 patients without endometriosis were implanted into 4 mice simultaneously at day 0 (Figure S1B). Then, mice were divided randomly into 4 groups: groups 1 and 3 (vehicle, controls) and groups 2 and 4 (CGP049090) (Sigma-Aldrich). Intraperitoneal injection of CGP049090 (2 mg/kg/day, once a day) (Sigma-Aldrich) or vehicle was started on day 7 (groups 1 and 2) or day 14 (groups 3 and 4) after endometrial tissue implantation and continued for 14 days (Figure S1B). Then, mice were sacrificed on day 21 (groups 1 an 2) or day 28 (groups 3 and 4) for collection of endometriotic implants. Intraperitoneal injection was performed under isoflurane anesthesia (Figure S1B). The dose and duration of treatment with CGP049090 was determined during preliminary experiments (Text S2).

#### d) Histology

Endometriotic implants were collected, fixed in 10% formalin-acetic acid, and embedded in paraffin for histopathological examination. Paraffin-embedded tissue sections were stained with hematoxylin and eosin, Masson Trichrome, or Sirius Red, according to common protocols [19]. Masson Trichrome staining and Sirius Red staining detect collagen fibrils that are deposited in the matrix [19]. To objectively quantify the severity of fibrosis in sections of endometriotic implants stained for collagen with Sirius Red or Masson Trichrome stains, we used a computerized image analysis system as previously described [5]. Several parameters were computed per sample for Sirius Red or Masson Trichrome stains in endometriotic implants: the percentage of stained surface (compared with the counterstained surface), the mean staining intensity, and the staining score (percentage of stained surface × mean staining intensity). The entire field of the endometriotic implant in each section was analyzed for Sirius Red or Masson Trichrome stains.

### Statistical analysis

The STATA program version 12 (StataCorp, College Station, Texas, USA) was used for statistical analysis. Comparisons between different groups were made using one-way analysis of variance (ANOVA) following Scheffé’s method, the Mann-Whitney *U* test, or the Wilcoxon matched pairs signed-ranks test. Statistical significance was defined as *P* < 0.05.

## Results

### Effects of β-Catenin siRNA on αSMA, Col-I, FN, and CTGF

**Figure 1:**
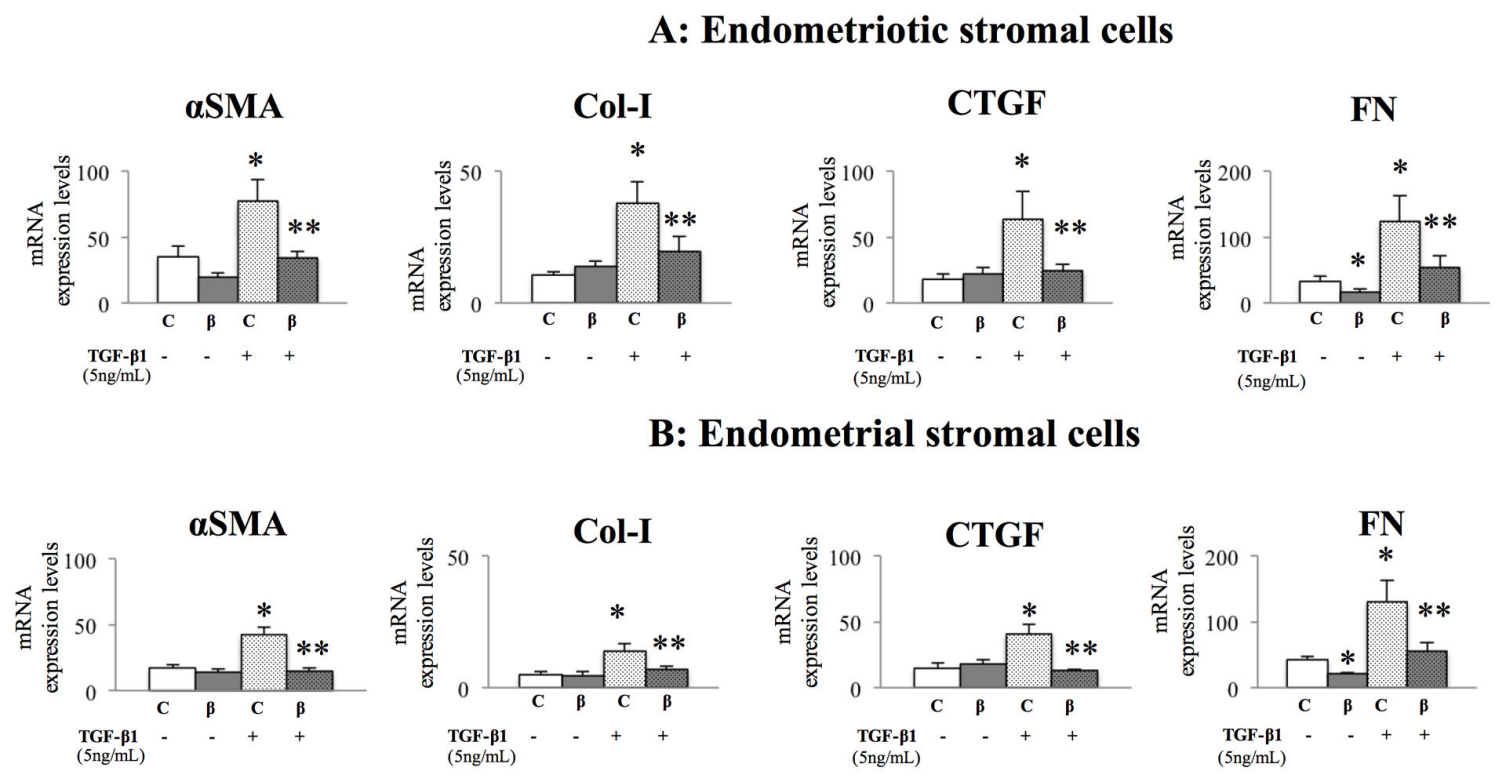
Effects of β-catenin siRNA on fibrotic markers in endometrial and endometriotic stromal cells from patients with endometriosis. Effects of β-catenin siRNA on the mRNA expression of *αSMA*, *Col-I*, *CTGF*, and *FN* in endometriotic (A) and endometrial (B) stromal cells with or without TGF-β1 stimulation. C: control siRNA-transfected cells; ß: ß-catenin siRNA-transfected cells. *: p<.05 versus control (C) cells without TGF-β1 stimulation. **: p<.05 versus control (C) cells with TGF-β1 stimulation. Numerical values are presented as the mean + SEM. Expression levels of *αSMA*, *Col-I*, *CTGF*, and *FN* mRNA are given relative to the expression level of the reference gene, *GAPDH*. Endometriotic stromal cells (n=10), endometrial stromal cells (n=10).

β-Catenin siRNA lowered β-catenin mRNA and protein expression by approximately 90%–95% and 60%, respectively, as previously reported [7]. Additionally, β-Catenin siRNA significantly lowered *FN* mRNA, whereas the expression of *αSMA*, *Col-I*, and *CTGF* transcripts was not altered by β-catenin siRNA, compared to control transfection, in both endometriotic (Figure 1A) and endometrial (Figure 1B) stromal cells. TGF-β1 stimulation increased the expression of *αSMA*, *Col-I*, *CTGF*, and *FN* transcripts, and this effect was significantly attenuated by β-catenin siRNA in endometriotic (Figure 1A) and endometrial (Figure 1B) stromal cells. The expression of hyaluronidase-2 (a non–Tcf/β-catenin or –TGF-β1 target gene, used as a negative control) was not altered by β-catenin siRNA with or without TGF-β1 stimulation (Figures S2A and S2B).

### Effects of PKF 115-584 and CGP049090 on αSMA, Col-I, FN, and CTGF

**Figure 2:**
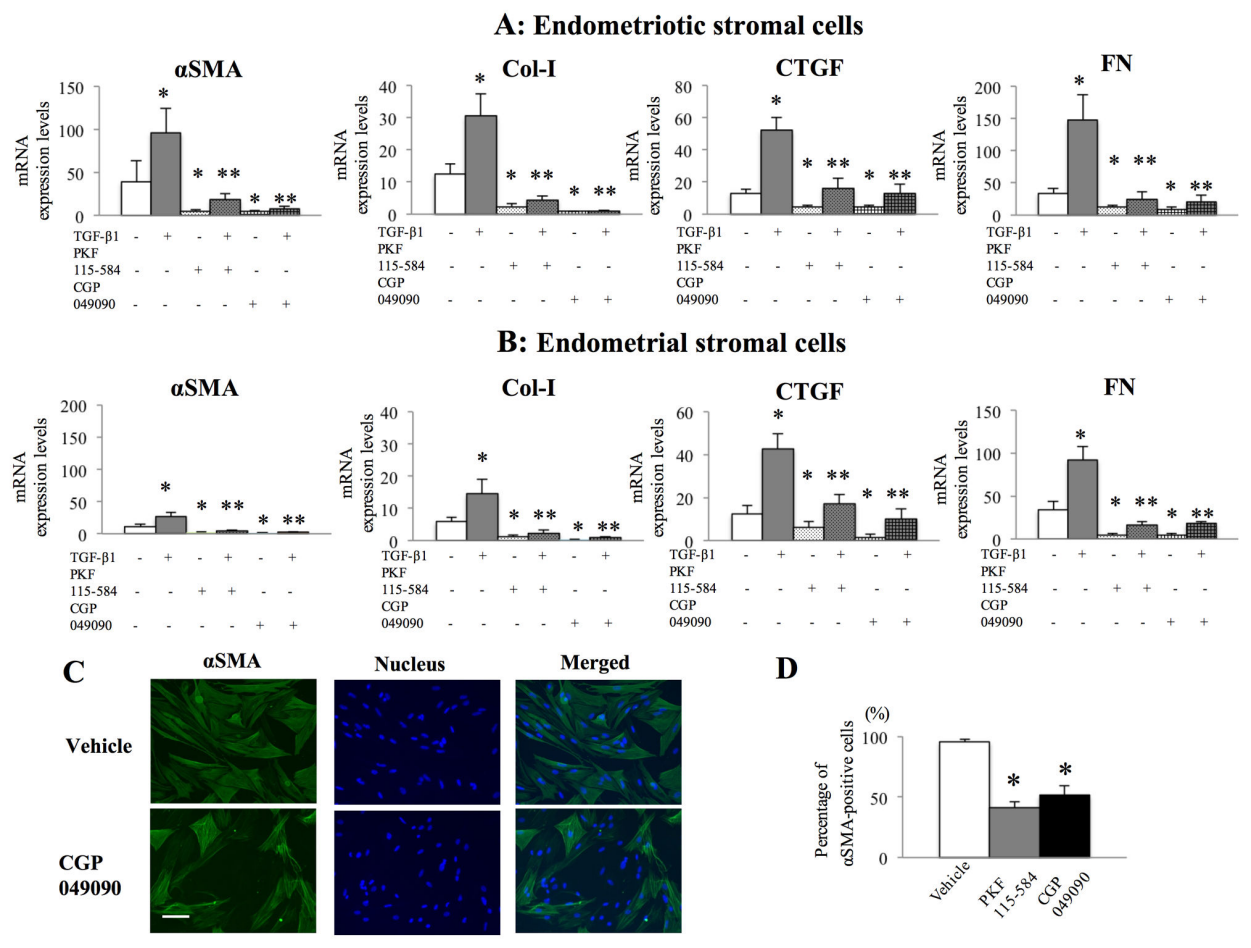
Effects of small-molecule antagonists of the Tcf/β-catenin complex (PKF 115-584 and CGP049090) on fibrotic markers in endometrial and endometriotic stromal cells from patients with endometriosis. A, B: Effects of small-molecule antagonists of the Tcf/β-catenin complex (PKF 115-584 and CGP049090) on the mRNA expression of *αSMA*, *Col-I*, *CTGF*, and *FN* in endometriotic (A) (n=10) and endometrial (B) (n=10) stromal cells with or without TGF-β1 stimulation. *: p<.05 versus vehicle-treated controls without TGF-β1 stimulation. **: p<.05 versus vehicle-treated controls with TGF-β1 stimulation. C: Representative photomicrographs of endometriotic stromal cells after treatment with vehicle or CGP049090 (6.25 µM) for 24 h and immunostained for αSMA (green) and nuclei (blue). Scale bar, 100 μm. D: Percentage of αSMA-positive endometriotic stromal cells (n=10) after treatment with vehicle, PKF 115-584 (6.25 µM), or CGP049090 (6.25 µM) for 24 h. *: p<.05 versus vehicle-treated controls. Numerical values are presented as the mean + SEM. Expression levels of *αSMA*, *Col-I*, *CTGF*, and *FN* mRNA are given relative to the expression level of the reference gene, *GAPDH*.

Treatment with small-molecule antagonists of the Tcf/β-catenin complex (PKF 115-584 and CGP049090) significantly decreased the expression of *αSMA*, *Col-I*, *CTGF*, and *FN* mRNAs in both endometriotic (Figure 2A) and endometrial (Figure 2B) stromal cells. In addition, treatment with PKF 115-584 and CGP049090 significantly attenuated the TGF-β1-dependent increase in the expression of these genes in both endometriotic (Figure 2A) and endometrial (Figure 2B) stromal cells. As a negative control, the expression of hyaluronidase-2 was not altered by treatment with small-molecule antagonists of the Tcf/β-catenin complex with or without TGF-β1 stimulation (Figures S2C and S2D). Immunofluorescence staining showed that endometriotic stromal cells had clearly visible αSMA-positive stress fibers (Figure 2C). Treatment with PKF 115-584 and CGP049090 significantly decreased the percentage of αSMA-positive endometriotic stromal cells compared to vehicle-treated controls (Figure 2D).

### Effects of PKF 115-584 and CGP049090 on collagen gel contraction

**Figure 3:**
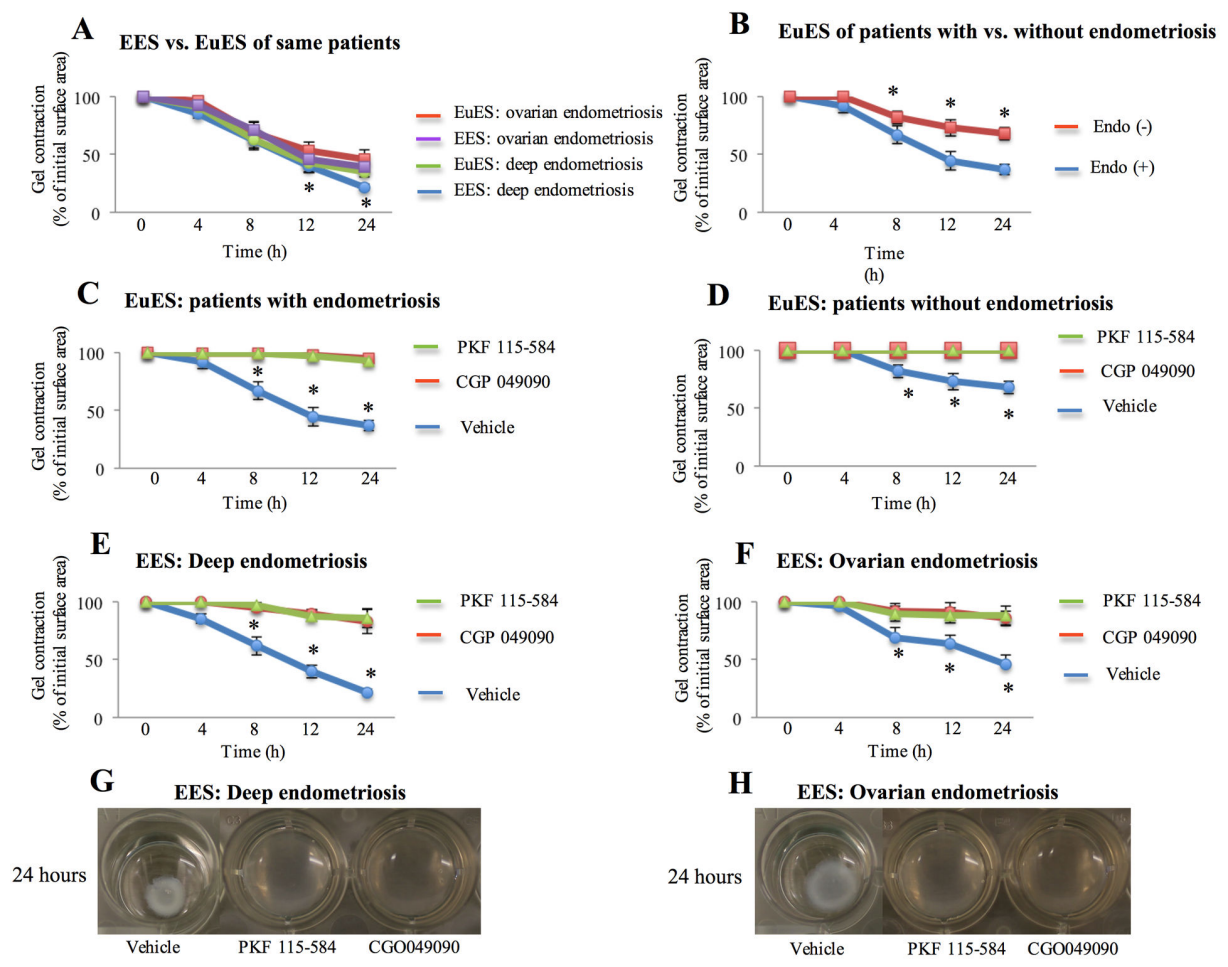
Effects of small-molecule antagonists of the Tcf/β-catenin complex (PKF 115-584 and CGP049090) on stromal cell-mediated collagen gel contraction. A–F: Collagen gel contraction at 0, 4, 8, 12, and 24 h in matched endometrial and endometriotic stromal cells from the same patient (A), *: p<.05: EES:deep endometriosis versus EES: ovarian endometriosis, endometrial stromal cells of patients with and without endometriosis (B), *: p<.05: versus EuEs: patients with endometriosis, treated and vehicle-treated endometrial stromal cells of patients with (C), *: p<.05: versus treated EuEs, and without endometriosis (D), *: p<.05: versus treated EuEs, treated and vehicle-treated endometriotic stromal cells (E, F), *: p<.05: versus treated EES, and G, H: Representative photomicrographs of contracted gels taken at 24 h in endometriotic stromal cells with and without treatment. Numerical values are presented as the mean + SEM. EES: endometriotic stromal cells; EuES: endometrial stromal cells. Endo (-): endometrium of patients without endometriosis. Endo (+): Endometrium of patients with endometriosis. EuES: ovarian endometriosis (n=10); EES: ovarian endometriosis (n=10). EuES: deep endometriosis (n=10); EES: deep endometriosis (n=10). EuES: patients with endometriosis (n=20); EuES: patients without endometriosis (n=10).

There was no significant difference in stromal cell-mediated collagen gel contraction between endometriotic tissues and matched eutopic endometrial tissues from the same patient with endometriosis during a 24-h period (Figure 3A). However, stromal cell-mediated contraction of collagen gels at 8, 12, and 24 h was significantly higher in the endometrium of patients with endometriosis compared to that of patients without endometriosis (Figure 3B). In addition, endometriotic stromal cell-mediated contraction of collagen gels at 12 and 24 h was significantly higher in deep infiltrating endometriosis than in ovarian endometriosis (Figure 3A). Both endometriotic and endometrial stromal cell-mediated contraction of collagen gels was significantly decreased by treatment with inhibitors of Tcf/β-catenin complex as compared to that of untreated cells (Figures 3C, 3D, 3E, 3F, 3G and 3H).

### Wnt3a-induced stimulation of profibrotic responses in endometrial stromal cells

**Figure 4:**
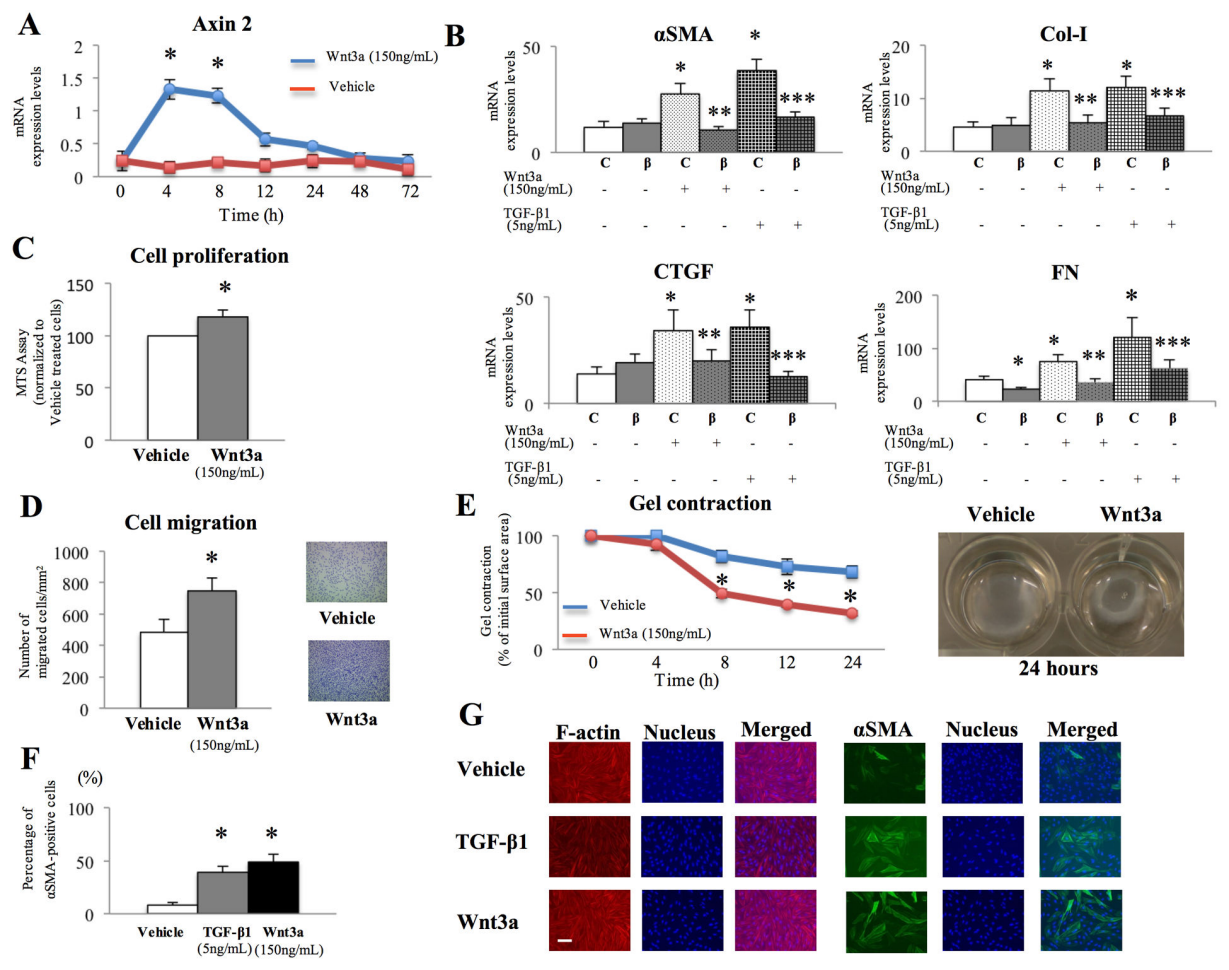
Effects of Wnt3a treatment on profibrotic responses in endometrial stromal cells of patients without endometriosis. A: Axin2 mRNA expression in endometrial stromal cells treated with vehicle or Wnt3a (150 ng/mL) for 0, 4, 8, 12, 24, 48, or 72 h (n=5). *: p<.05 versus vehicle-treated controls. B: Effects of β-catenin siRNA on the mRNA expression of *αSMA*, *Col-I*, *CTGF*, and *FN* in endometrial stromal cells treated with vehicle, Wnt3a (150 ng/mL), or TGF-β1 (5 ng/mL) for 24 h (n=10). C: control siRNA-transfected cells; ß: ß-catenin siRNA-transfected cells. *: p<.05 versus control (C) cells without stimulation. **: p<.05 versus control (C) cells with Wnt3a stimulation. ***: p<.05 versus control (C) cells with TGF-β1 stimulation. C: Cell proliferation of endometrial stromal cells treated with vehicle or Wnt3a (150 ng/mL) for 72 h (n=5). *: p<.05 versus vehicle-treated controls. D: Cell migration of endometrial stromal cells treated with vehicle or Wnt3a (150 ng/mL) for 72 h (n=5). *: p<.05 versus vehicle-treated controls. E: Collagen gel contraction of stromal cells treated for 24 h with vehicle or Wnt3a (150 ng/mL) (n=5), and representative photomicrographs of contracted gels taken at 24 h in stromal cells treated with vehicle or Wnt3a (150 ng/mL). *:p<.05 versus vehicle-treated controls. F: Percentage of αSMA-positive cells treated for 24 h with vehicle, Wnt3a (150 ng/mL), or TGF-β1 (5 ng/mL) (n=5). *:p<.05 versus vehicle-treated controls. G: Representative photomicrographs of endometrial stromal cells from the same patient after treatment with vehicle, Wnt3a (150 ng/mL), or TGF-β1 (5 ng/mL) for 24 h and stained for f-actin (red) or αSMA (green) and nuclei (blue). Scale bar, 100 μm. Numerical values are presented as the mean + SEM. Expression levels of axin2, *αSMA*, *Col-I*, *CTGF*, and *FN* mRNAs are given relative to the expression of the reference gene, *GAPDH*.

Our previous study suggested that the Wnt/β-catenin signaling pathway may be aberrantly activated in the endometrium of patients with endometriosis [5]. Thus, in the present study, we attempted to activate the Wnt/β-catenin signaling pathway by recombinant Wnt3a in the endometrium of patients without endometriosis. Wnt3a treatment in endometrial stromal cells increased the mRNA expression of axin2, an early immediate target of canonical Wnt signaling, compared with vehicle-treated cells (Figure 4A). Moreover, Wnt3a treatment in endometrial stromal cells significantly increased cell proliferation (Figure 4C) and migration (Figure 4D) as well as endometrial stromal cell-mediated collagen contraction (Figure 4E). Collagen gel contraction in Wnt3a-treated endometrial cells was comparable with that of the endometrium of patients with endometriosis. Wnt3a treatment also significantly increased the mRNA expression of the fibrotic marker genes *αSMA*, *COL-*I, FN, and *CTGF*, and this effect was attenuated by transfection with β-catenin siRNA (Figure 4B). However, Wnt3a treatment had no significant effect on the expression of these genes in the endometrium of patients with endometriosis (Figure S3). On the contrary, no significant difference was noted in the TGF-β1-dependent increase of expression of these genes between patients with and without endometriosis (Figure S4). Both Wnt3a- and vehicle-treated endometrial stromal cells had clearly visible f-actin-positive stress fibers (Figure 4G). When cultured on a plastic substrate in the presence of fetal calf serum, all fibroblasts acquire stress fibers that express cytoplasmic actins [20]. Wnt3a-treated endometrial cells had clearly visible αSMA-positive stress fibers, while few vehicle-treated cells displayed expression of αSMA in their stress fibers (Figure 4G). The stimulatory effects of Wnt3a on αSMA-positive stress fibers in endometrial stromal cells were comparable with those of TGF-β1 (Figure 4F and 4G).

### Mouse model of endometriosis

The present in vitro experiments detected no significant difference in the effects of the 2 small-molecule antagonists of the Tcf/β-catenin complex (PKF 115-584 and CGP049090) on either the expression of fibrotic markers or collagen gel contraction. Thus, we assessed only the effects of CGP049090 in the present in vivo mouse experiments. All of the mice in the present study developed endometriotic lesions, showing typical chsaracteristics of endometriosis, with glandular structures and stroma. To monitor the overall well being of the mice treated with CGP049090 or vehicle, the mice were monitored daily, and body weights recorded. Over the experimental period, all mice survived, and there was no significant difference in growth rates between treated and untreated control mice.

**Figure 5:**
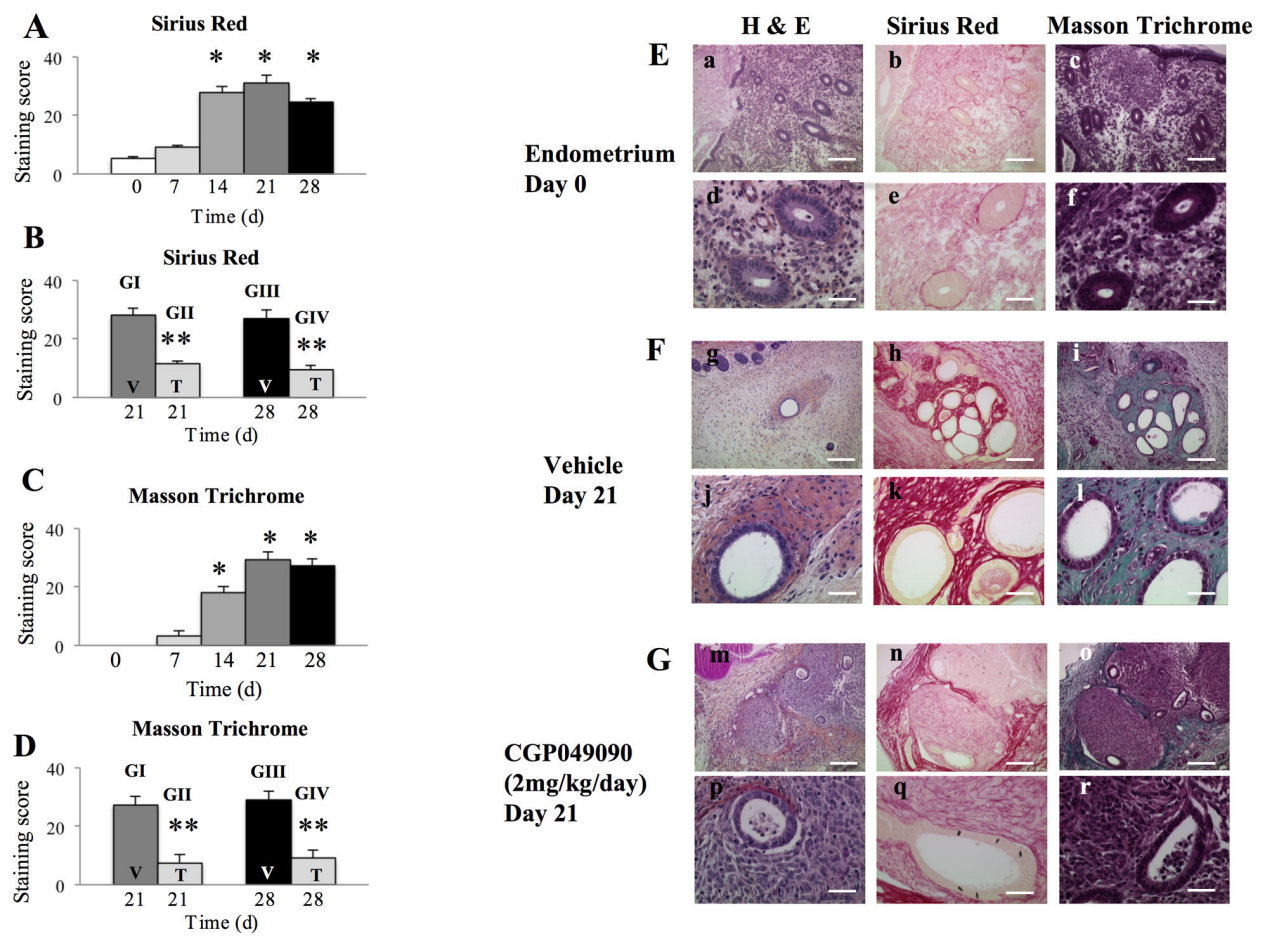
Effects of CGP049090 treatment on fibrosis in a mouse endometriosis model. A-D: Staining score for Sirius Red (A, B) or Masson Trichrome (C, D) staining in a time course study of fibrosis development (on days 0, 7, 14, 21, and 28) (A, C) and vehicle-treated and CGP049090 (2 mg/kg)-treated mice (Groups I and II: treated once a day between days 7 and 21 for 14 days; Groups III and IV: treated once a day between days 14 and 28 for 14 days) (B, D). *: p<.05 versus endometrium on day 0 and endometriotic implants on day 7. **: p<.05 versus vehicle-treated mice. Day 0 (endometrium) (n=10), Days 7 (n=10), 14 (n=10), 21 (n=10), or 28 (n=10). V: vehicle-treated mice; T: CGP049090 (2 mg/kg)-treated mice. G1: Group I (n=10), GII: Group II (n=10), GIII: Group III (n=10), GIV: Group IV (n=10). E-G: Representative photomicrographs of the endometrium stained with hematoxylin and eosin, Sirius Red, or Masson Trichrome on day 0 (E), or of endometriotic implants of mice treated for 14 days with vehicle alone (F) or with CGP049090 (G) at day 21. Scale bars (a-c, g-i, m-o: 200 μm; d-f, j-l, p-r: 50 μm).

When the time course of fibrosis development was evaluated by Sirius Red and Masson Trichrome staining, there were no significant differences in staining scores for either stain between the endometrium on day 0 and endometriotic implants on day 7 (Figure 5A and 5C). However, scores for these 2 stains were significantly increased in endometriotic implants on day 14, and these endometriotic implants maintained higher scores until day 28, as compared to those of the endometrium on day 0 (Figure 5A and 5C). Thus, we attempted to evaluate the effects of CGP049090 treatment on the fibrosis of endometriotic implants, when fibrosis was not yet evident (on day 7) and when fibrosis was already established (on day 14).

When treatment was started on day 7, the staining scores for both Sirius Red and Masson Trichrome stains were significantly lower in treated mice than in untreated mice (Figures 5B, 5D, 5E, 5F and 5G). Scores for Sirius Red and Masson Trichrome staining in endometriotic implants of treated mice were similar to those of the endometrium on day 0 and endometriotic implants on day 7 (Figures S5A and S5B). When treatment was started on day 14, scores for both Sirius Red and Masson Trichrome staining were significantly lower in treated mice than in untreated mice (Figure 5B and 5D). In addition, scores for Sirius Red and Masson Trichrome staining in endometriotic implants of treated mice were significantly lower than those in endometriotic implants on day 14 and were similar to those of the endometrium on day 0 and endometriotic implants on day 7 (Figures S5A and S5B).

## Discussion

The present study demonstrated that the molecular and cellular mechanisms known to be involved in fibrogenesis were inhibited by targeting the Wnt/β-catenin pathway in endometrial and endometriotic stromal cells in vitro. Treatment with small-molecule antagonists of the Tcf/β-catenin complex (PKF 115-584 and CGP049090) significantly decreased the expression of genes known to be involved in fibrogenesis in endometrial and endometriotic stromal cells. In addition, treatment with the small-molecule antagonists of the Tcf/β-catenin complex significantly decreased endometrial and endometriotic stromal cell–mediated contraction of collagen gels. Our previous study demonstrated that treatment with a small-molecule antagonist of the Tcf/β-catenin complex could significantly decrease cell proliferation and migration of endometrial and endometriotic stromal cells [7]. Fibroblast migration, proliferation, and collagen contraction are critical aspects of fibrogenesis [21]. The present findings and our previous findings suggested that treatment with small-molecule antagonists of the Tcf/β-catenin complex may inhibit these critical aspects of fibrogenesis in endometriosis.

In the present study, we also found that Wnt3a treatment in the endometrium of patients without endometriosis significantly increased stromal cell proliferation and migration, stromal cell-mediated contraction of collagen gels, and expression of fibrotic marker genes. Stromal cell-mediated collagen gel contraction in cells from patients without endometriosis was significantly lower than that from patients with endometriosis. Wnt3a treatment in stromal cells of patients without endometriosis increased collagen gel contraction to a level comparable with that of the endometrium from patients with endometriosis. Wnt3a-treated endometrial stromal cells had clearly visible αSMA-positive stress fibers, demonstrating that Wnt3a induced myofibroblast differentiation [20]. The present findings suggested that the aberrant activation of the Wnt/β-catenin pathway may be involved in the molecular and cellular mechanisms of fibrogenesis in endometriosis.

The present study observed that both β-catenin siRNA and treatment with small-molecule antagonists of the Tcf/ß-catenin complex significantly attenuated TGF-β1-induced fibrotic markers in endometrial and endometriotic stromal cells. TGF-β is a key mediator in fibrosis [21-23]. A recent study demonstrated that canonical Wnt signaling is necessary for TGF-β-mediated fibrosis, highlighting a key role for the interaction of both pathways in the pathogenesis of fibrotic diseases [16]. The present findings may support this. Moreover, there is evidence that TGF-β1 is involved in the pathophysiology of endometriosis [24]. A recent study demonstrated that host-derived TGF-β1 deficiency suppressed endometriotic lesion development in a mouse model [25]. Labbé et al. demonstrated the complex, intertwined nature of the TGF-β and Wnt/β-catenin pathways in mammary and intestinal tumorigenesis [26]. Further studies to investigate the interaction between the two signaling pathways in endometriosis may provide further insights into the pathophysiology of endometriosis and facilitate the development of novel therapeutic strategies for endometriosis.

The present animal experiments suggested that treatment with CGP049090, a small-molecule antagonist of the Tcf/β-catenin complex, may prevent the progression of fibrosis during the initial development of endometriosis. More importantly, the present findings suggested that this compound may reverse established fibrosis. These findings are in accordance with a previous study conducted in a mouse model of bleomycin-induced pulmonary fibrosis [15]. Co-administration of ICG-001, a selective inhibitor of Wnt/β-catenin-CBP-dependent transcription, and bleomycin prevented fibrosis, and late administration was able to reverse established fibrosis [15]. These findings and our present findings support an important role for the Wnt/β-catenin signaling pathway in the pathogenesis of fibrosis.

## Conclusion

The present findings demonstrated that the molecular and cellular mechanisms known to be involved in fibrogenesis are inhibited by targeting the Wnt/β-catenin pathway in endometrial and endometriotic stromal cells in vitro. In addition, the present animal experiments suggested that targeting the Wnt/β-catenin pathway may prevent the progression of fibrosis and reverse established fibrosis in endometriosis. Aberrant activation of the Wnt/β-catenin pathway may be involved in mediating fibrogenesis in endometriosis. As the dose of PKF 115-584 and CGP049090 used in the present study may not be clinically achievable [27], PKF 115-584 and CGP049090 themselves might not be drug candidates for treatment and/or prevention of endometriosis. However, the present in vivo findings provide further support for the speculation that the Wnt/β-catenin signaling pathway may represent a novel therapeutic target for prevention and treatment of endometriosis. A study demonstrated that patients with the highest pre-operative pain scores display higher proportions of nerve encapsulation in fibrosis of deep infiltrating endometriosis [28]. Further preclinical studies are necessary to investigate whether targeting the Wnt/β-catenin pathway in endometriosis could relieve pain symptoms. However, one major concern in targeting the Wnt/β-catenin pathway is the potential for side effects in tissues that require the Wnt/β-catenin signaling for physiological cell renewal [29]. Further studies are required to pay close attention to potential side effects of in vivo use of the Wnt/β-catenin pathway inhibitors in patients with endometriosis.

## Supporting Information

Text S1 **Collagen gel contraction assay.**
(DOCX)

Text S2 **CGP049090 treatment.**
(DOCX)

Figure S1 **Experimental design for the mouse experiment.** A: Time course study of fibrosis development.
B: Effects of CGP049090 treatment on the fibrosis of endometriotic implants.
(TIF)

Figure S2 **Effects of β-catenin siRNA and small-molecule antagonists of the Tcf/β-catenin complex (PKF 115-584 and CGP049090) on the mRNA expression of hyaluronidase-2 in endometrial and endometriotic stromal cells from patients with endometriosis.** A, B: Effects of β-catenin siRNA on the mRNA expression of hyaluronidase-2 in endometriotic (A) (n=10) and endometrial (B) (n=10) stromal cells with or without TGF-β1 stimulation.
C, D: Effects of small-molecule antagonists of the Tcf/β-catenin complex (PKF 115-584 and CGP049090) on the mRNA expression of hyaluronidase-2 in endometriotic (C) (n=10) and endometrial (D) (n=10) stromal cells with or without TGF-β1 stimulation.
Numerical values are presented as the mean + SEM. Expression levels of hyaluronidase-2 mRNA are given relative to the expression level of the reference gene, *GAPDH*.
C: control siRNA-transfected cells; ß: ß-catenin siRNA-transfected cells.
(TIF)

Figure S3 **Effects of Wnt3a on fibrotic markers in endometrial stromal cells from patients with versus without endometriosis.** Effects of Wnt3a on the mRNA expression of *αSMA*, *Col-I*, *CTGF*, and *FN* in endometrial stromal cells from patients with (n=10) and without (n=10) endometriosis.
Cells were treated with vehicle or Wnt3a (150 ng/mL) for 24 h.
C: control siRNA-transfected cells; ß: ß-catenin siRNA-transfected cells.
*: p<.05 versus control (C) cells without Wnt3a stimulation.
**: p<.05 versus control (C) cells with Wnt3a stimulation.
Numerical values are presented as the mean + SEM. Expression levels of *αSMA*, *Col-I*, *CTGF*, and *FN* mRNA are given relative to the expression level of the reference gene, *GAPDH*.
Endo (+): Endometrium of patients with endometriosis.
Endo (-): endometrium of patients without endometriosis.
(TIF)

Figure S4 **Effects of TGF-β1 on fibrotic markers in endometrial stromal cells from patients with versus without endometriosis.** Effects of TGF-β1 on the mRNA expression of *αSMA*, *Col-I*, *CTGF*, and *FN* in endometrial stromal cells from patients with (n=10) and without (n=10) endometriosis.
Cells were treated with vehicle or TGF-β1 (5 ng/mL) for 24 h.
C: control siRNA-transfected cells; ß: ß-catenin siRNA-transfected cells.
Numerical values are presented as the mean + SEM. Expression levels of *αSMA*, *Col-I*, *CTGF*, and *FN* mRNA are given relative to the expression level of the reference gene, *GAPDH*.
Endo (+): Endometrium of patients with endometriosis.
Endo (-): endometrium of patients without endometriosis.
(TIF)

Figure S5 **Staining score for Sirius Red or Masson Trichrome in vehicle-treated and CGP049090 (2 mg/kg)-treated mice.** A: Sirius Red, B: Masson Trichrome.
In vehicle-treated mice: Day 0 (endometrium) (n=10), Days 7 (n=10), 14 (n=10), 21 (n=10), or 28 (n=10). CGP049090-treated mice: Days 7 (n=10) or 28 (n=10).
*: p<.05 versus non-treated mice at 14 days.
(TIF)

Table S1 **Clinical characteristics of patients.**
(DOCX)

Table S2 **Sequences of the primers used for mRNA quantitation by real-time RT-PCR.**
(DOCX)
