# Supplementary material for: Involvement of the Wnt/β-Catenin Signaling Pathway in the Cellular and Molecular Mechanisms of Fibrosis in Endometriosis
Source: PLoS One. 2013 Oct 4;8(10):e76808. doi: 10.1371/journal.pone.0076808 (PMC3790725; doi:10.1371/journal.pone.0076808)
Supplement: Text S1 — Collagen gel contraction assay. (DOCX) [file pone.0076808.s001.docx]

**Text S1**

**Collagen gel contraction assay**

During preliminary experiments, stromal cell-mediated collagen gel contraction was assessed at different concentrations of charcoal-stripped FBS (0%, 2%, or 10%) in culture media. There were no significant differences in endometriotic stromal cell-mediated collagen gel contraction among cells cultured in media with 0%, 2%, and 10% charcoal-stripped FBS. However, endometrial stromal cell-mediated collagen gel contraction in serum-free culture media was significantly lower than those in culture media with 2% or 10% charcoal-stripped FBS. In the present study, we evaluated the effects of PKF 115-584 and CGP049090 on collagen gel contraction in culture media with 2% FBS to minimize the influence of FBS on the assay. Furthermore, during preliminary experiments, contraction was assessed at 4, 6, 12, 24, and 48 h. A time-dependent augmentation of contraction was observed until 24 h; however, only a small additional contraction was observed between 24 and 48 h. Thus, in the present study, we assessed collagen gel contraction at 4, 6, 12, and 24 h.
