# Supplementary material for: Involvement of the Wnt/β-Catenin Signaling Pathway in the Cellular and Molecular Mechanisms of Fibrosis in Endometriosis
Source: PLoS One. 2013 Oct 4;8(10):e76808. doi: 10.1371/journal.pone.0076808 (PMC3790725; doi:10.1371/journal.pone.0076808)
Supplement: Text S2 — CGP049090 treatment. (DOCX) [file pone.0076808.s002.docx]

**Text S2**

**CGP049090 treatment**

During our preliminary experiments, we evaluated 2 different dosages of CGP049090 (1 and 2 mg/kg) and 2 different durations of treatment (once a day or once every 2 days for 14 days) using 3 mice for each treatment protocol. Each treatment was started at 1 week after endometrial tissue implantation. For all protocols, no animals lost weight, and all animals were healthy during treatment. We selected a dose of 2 mg/kg/day and once a day for 14 days for the present study because this protocol resulted in reduced fibrosis as evaluated by Sirius Red staining than other treatment protocols.
