# Supplementary figures and images for: Involvement of the Wnt/β-Catenin Signaling Pathway in the Cellular and Molecular Mechanisms of Fibrosis in Endometriosis
Source: PLoS One. 2013 Oct 4;8(10):e76808. doi: 10.1371/journal.pone.0076808 (PMC3790725; doi:10.1371/journal.pone.0076808)

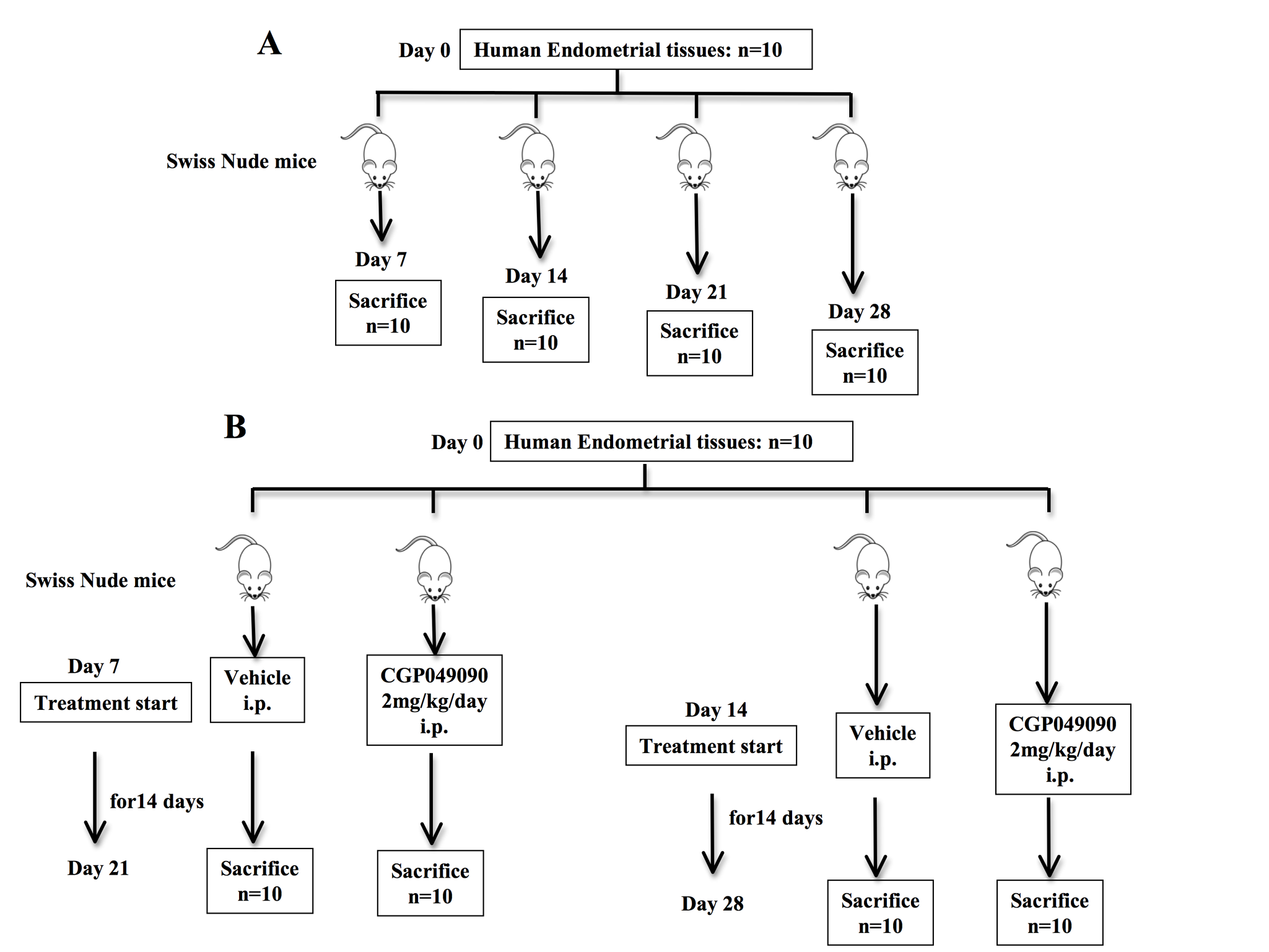

Supplement: Figure S1 — Experimental design for the mouse experiment. A: Time course study of fibrosis development. B: Effects of CGP049090 treatment on the fibrosis of endometriotic implants. (TIF) [file pone.0076808.s003.tif]

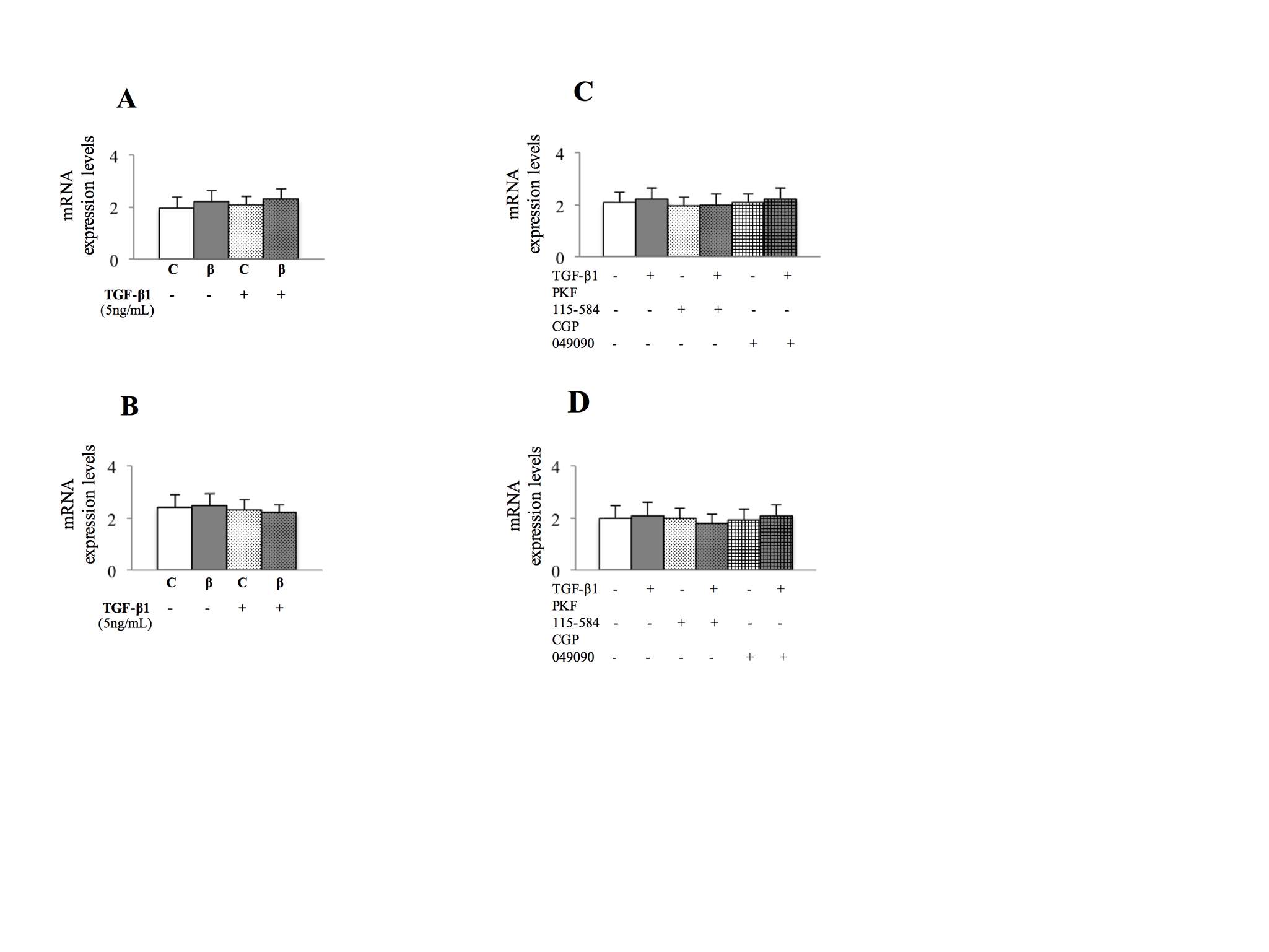

Supplement: Figure S2 — Effects of β-catenin siRNA and small-molecule antagonists of the Tcf/β-catenin complex (PKF 115-584 and CGP049090) on the mRNA expression of hyaluronidase-2 in endometrial and endometriotic stromal cells from patients with endometriosis. A, B: Effects of β-catenin siRNA on the mRNA expression of hyaluronidase-2 in endometriotic (A) (n=10) and endometrial (B) (n=10) stromal cells with or without TGF-β1 stimulation. C, D: Effects of small-molecule antagonists of the Tcf/β-catenin complex (PKF 115-584 and CGP049090) on the mRNA expression of hyaluronidase-2 in endometriotic (C) (n=10) and endometrial (D) (n=10) stromal cells with or without TGF-β1 stimulation. Numerical values are presented as the mean + SEM. Expression levels of hyaluronidase-2 mRNA are given relative to the expression level of the reference gene, GAPDH. C: control siRNA-transfected cells; ß: ß-catenin siRNA-transfected cells. (TIF) [file pone.0076808.s004.tif]

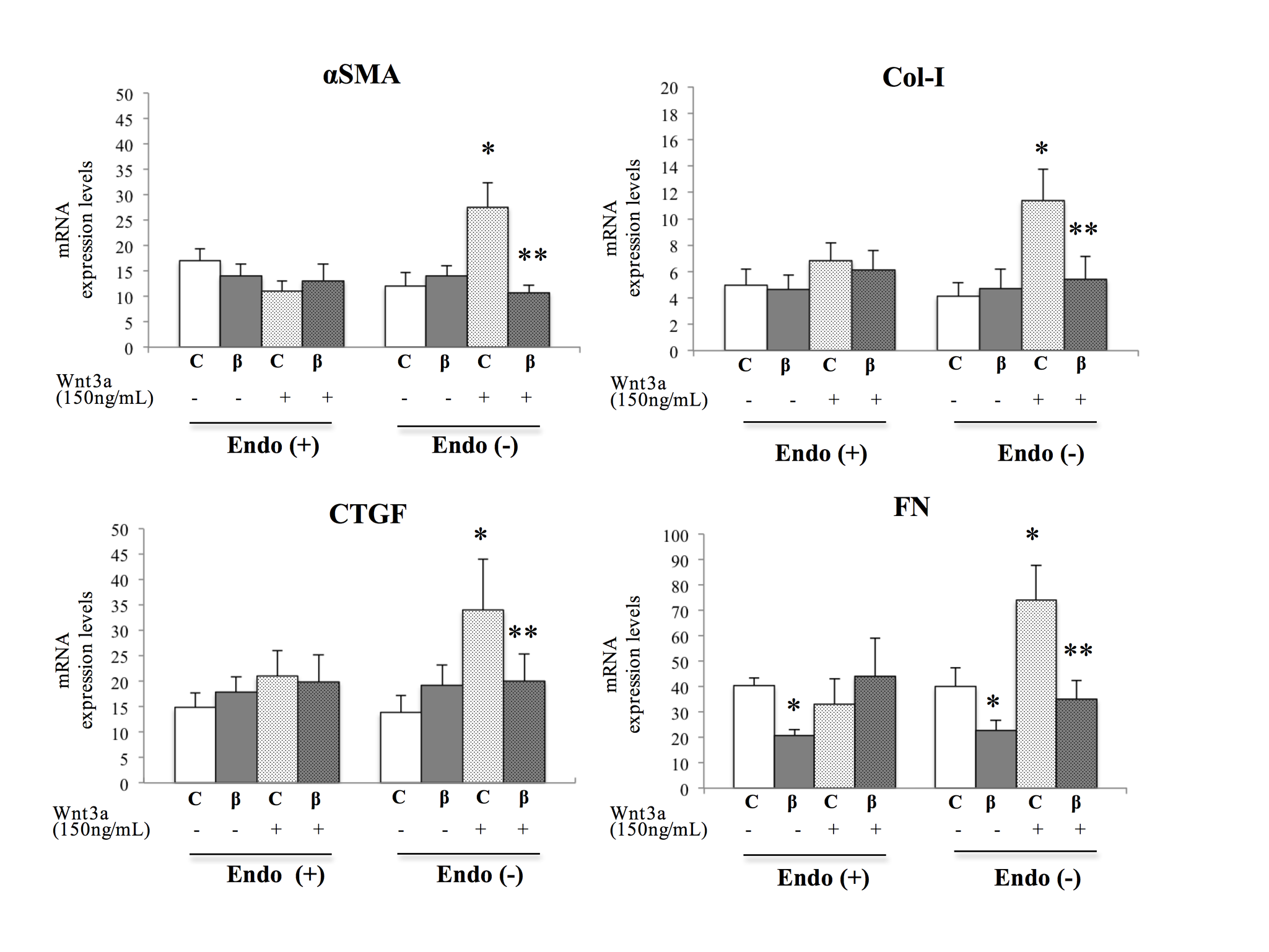

Supplement: Figure S3 — Effects of Wnt3a on fibrotic markers in endometrial stromal cells from patients with versus without endometriosis. Effects of Wnt3a on the mRNA expression of αSMA, Col-I, CTGF, and FN in endometrial stromal cells from patients with (n=10) and without (n=10) endometriosis. Cells were treated with vehicle or Wnt3a (150 ng/mL) for 24 h. C: control siRNA-transfected cells; ß: ß-catenin siRNA-transfected cells. *: p<.05 versus control (C) cells without Wnt3a stimulation. **: p<.05 versus control (C) cells with Wnt3a stimulation. Numerical values are presented as the mean + SEM. Expression levels of αSMA, Col-I, CTGF, and FN mRNA are given relative to the expression level of the reference gene, GAPDH. Endo (+): Endometrium of patients with endometriosis. Endo (-): endometrium of patients without endometriosis. (TIF) [file pone.0076808.s005.tif]

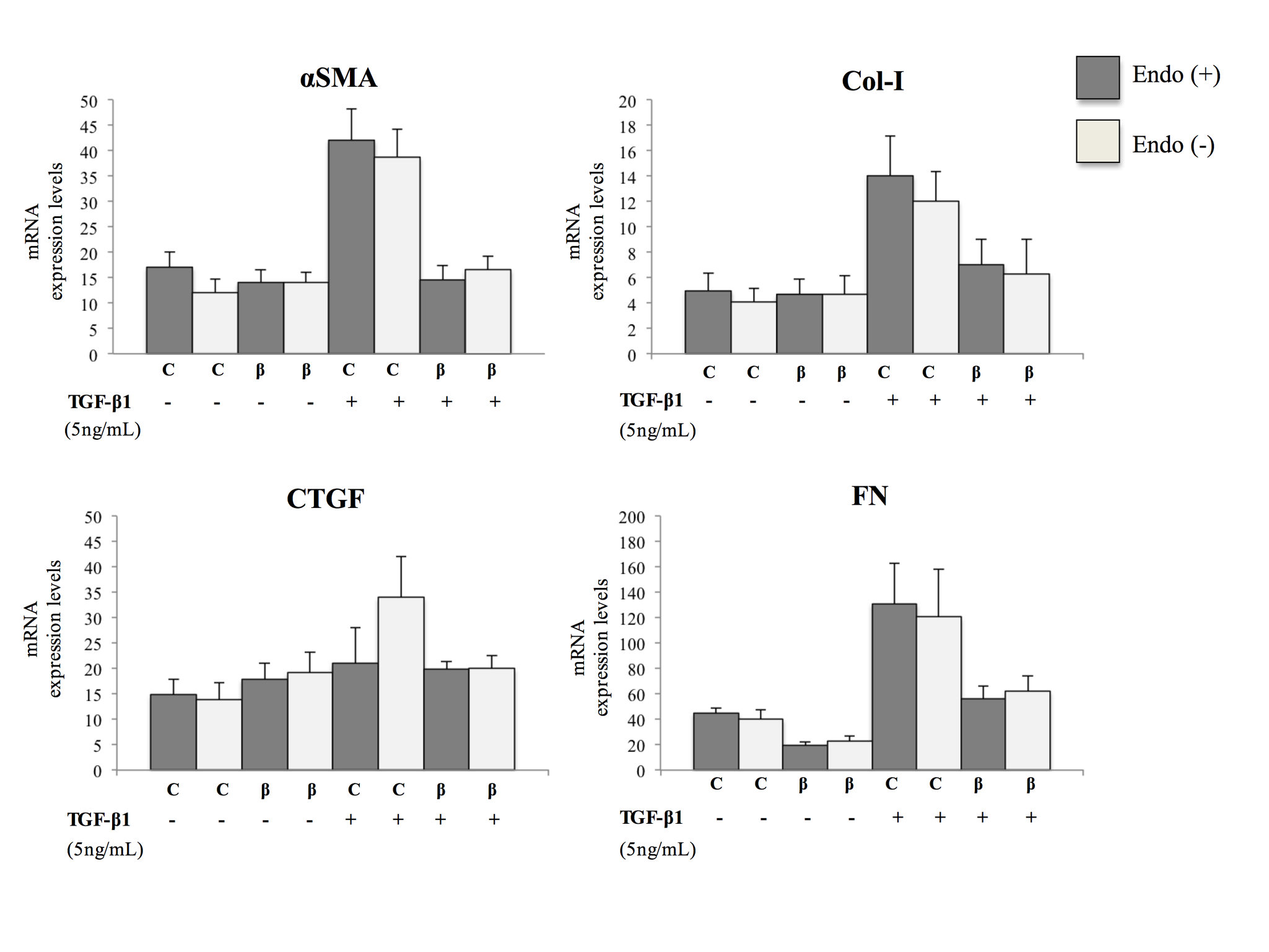

Supplement: Figure S4 — Effects of TGF-β1 on fibrotic markers in endometrial stromal cells from patients with versus without endometriosis. Effects of TGF-β1 on the mRNA expression of αSMA, Col-I, CTGF, and FN in endometrial stromal cells from patients with (n=10) and without (n=10) endometriosis. Cells were treated with vehicle or TGF-β1 (5 ng/mL) for 24 h. C: control siRNA-transfected cells; ß: ß-catenin siRNA-transfected cells. Numerical values are presented as the mean + SEM. Expression levels of αSMA, Col-I, CTGF, and FN mRNA are given relative to the expression level of the reference gene, GAPDH. Endo (+): Endometrium of patients with endometriosis. Endo (-): endometrium of patients without endometriosis. (TIF) [file pone.0076808.s006.tif]

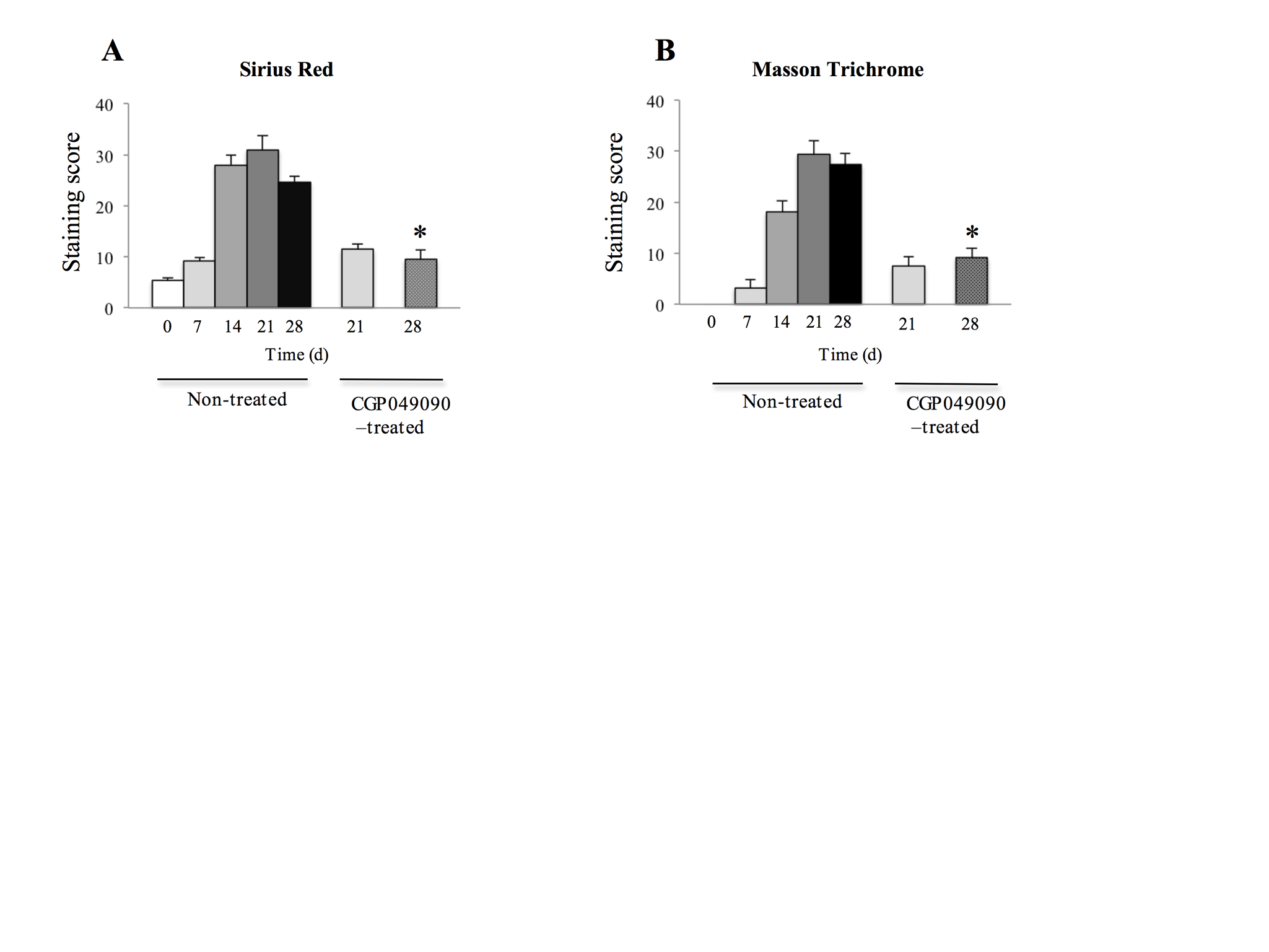

Supplement: Figure S5 — Staining score for Sirius Red or Masson Trichrome in vehicle-treated and CGP049090 (2 mg/kg)-treated mice. A: Sirius Red, B: Masson Trichrome. In vehicle-treated mice: Day 0 (endometrium) (n=10), Days 7 (n=10), 14 (n=10), 21 (n=10), or 28 (n=10). CGP049090-treated mice: Days 7 (n=10) or 28 (n=10). *: p<.05 versus non-treated mice at 14 days. (TIF) [file pone.0076808.s007.tif]
