# Supplementary material for: Involvement of the Wnt/β-Catenin Signaling Pathway in the Cellular and Molecular Mechanisms of Fibrosis in Endometriosis
Source: PLoS One. 2013 Oct 4;8(10):e76808. doi: 10.1371/journal.pone.0076808 (PMC3790725; doi:10.1371/journal.pone.0076808)
Supplement: Table S1 — Clinical characteristics of patients. (DOCX) [file pone.0076808.s008.docx]

**Table S1. Clinical characteristics of patients**

|  |  | | Endometriosis | | |  | | Uterine fibroma |  | Tubal  infertility |
| --- | --- | --- | --- | --- | --- | --- | --- | --- | --- | --- |
|  | DE | | | OE | | |  |  |  |  |
| No of cases | | 20 | | | 20 | |  | 14 |  | 16 |
| Age ^a^ | | 31.5  (21-36) | | | 31.0  (22-35) | |  | 32.0  (22-37) |  | 30.5  (21-37) |
| Parity ^a^ | | 0 (0-1) | | | 0 (0-1) | |  | 0 (0-1) |  | 0 (0-1) |
| rASRM stage^b^  I  II  III  IV | | 8  5  4  3 | | | 0  0  13  7 | |  |  |  |  |

^a^ Median (range)

^b^ Revised American Society for Reproductive Medicine classification (rASRM) (American Society for Reproductive Medicine, 1997).

DE: patients with deep endometriosis

OE: patients with ovarian endometriosis
