# Supplementary material for: Involvement of the Wnt/β-Catenin Signaling Pathway in the Cellular and Molecular Mechanisms of Fibrosis in Endometriosis
Source: PLoS One. 2013 Oct 4;8(10):e76808. doi: 10.1371/journal.pone.0076808 (PMC3790725; doi:10.1371/journal.pone.0076808)
Supplement: Table S2 — Sequences of the primers used for mRNA quantitation by real-time RT-PCR. (DOCX) [file pone.0076808.s009.docx]

**Table S2: Sequences of the primers used for mRNA quantitation by real-time RT-PCR**

| Gene |  | | Sense primers |  | | Antisense primers | |
| --- | --- | --- | --- | --- | --- | --- | --- |
| αSMA |  | 5´- GACCCTGAAGTACCCGATAGA-3´ | | |  | | 5´- GGGCAACACGAAGCTCATTG-3´ |
| Col-I |  | 5´- AGCCAGCAGATCGAGAACAT-3´ | | |  | | 5´- TCTTGTCCTTGGGGTTCTTG-3´ |
| CTGF | 111 | 5´- GGCCCAGACCCAACTATGATTA -3´ | | |  | | 5´- AGGAGGCGTTGTCATTGGTAAC -3´ |
| FN |  | 5´- TCGAGGAGGAAATTCCAATG-3´ | | |  | | 5´- ACACACGTGCACCTCATCAT-3´ |
| Axin-2 |  | 5´- TGAGCGGGATGCTTTGAAC-3´ | | |  | | 5´- ATCCTGTCTCTGTGCATTGCTG-3´ |
| ß-catenin |  | 5'-CGTTTGGCTGAACCATCA-3' | | |  | | 5'-TGAGGAGAACGCATGATAGCG-3' |
| Hyal-2 |  | 5´-TGTGAGCTTCCGTGTTCAG-3´ | | |  | | 5´-GTCTCCGTGCTTGTGGTGTA-3´ |
| GAPDH |  | 5'-TGCACCACCAACTGCTTAG-3' | | |  | | 5'-CTCTCGTTCACCTCGATCTTCA-3' |

αSMA: alpha smooth muscle actin

Col-I: Type I collagen

CTGF: connective tissue growth factor

FN: fibronection

Hyal-2: hyaluronidases 2

GAPDH: glyceraldehyde 3-phosphate dehydrogenase
